# Obesity-related proteins score as a potential marker of breast cancer risk

**DOI:** 10.1038/s41598-021-87583-3

**Published:** 2021-04-15

**Authors:** Sha Diao, Xueyao Wu, Xiaofan Zhang, Yu Hao, Bin Xu, Xu Li, Lulu Tian, Yunqi Miao, Xunying Zhao, Feng Ye, Jiayuan Li

**Affiliations:** 1 Department of Epidemiology and Health Statistics, West China School of Public Health and West China Fourth Hospital, Sichuan University, Chengdu, 610041 Sichuan China; 2 Department of Pharmacy, West China Second University Hospital, Sichuan University, Chengdu, 610041 Sichuan China; 3 Department of Clinical Research Management, West China Hospital, Sichuan University, Chengdu, 610041 Sichuan China; 4 Institute of Clinical Pathology, West China Hospital, Sichuan University, Chengdu, 610041 Sichuan China

**Keywords:** Cancer, Biomarkers, Risk factors

## Abstract

There is strong evidence to suggest that obesity-related proteins play a key role in pathways that are related to breast cancer. In this study, we aimed to establish a robust obesity-related protein score (ORPS) that could be used to assess breast cancer risk. Based on evidence from high-quality systematic reviews and population studies, we selected nine such proteins that are stable in vitro, and measured their circulating concentrations by ELISA in a case–control study conducted in Chengdu, Sichuan, China, with 279 breast cancer cases and 260 healthy controls. Two obesity-related protein scores (ORPS) were calculated using a three-step method, with linear-weighted summation, and the one with a larger area under the curve was chosen for further evaluation. As a result, ORPS (PS_5pre_ or PS_4post_) was positively correlated with breast cancer risk (premenopausal: OR_≤63 VS >63_ 3.696, 95% CI 2.025–6.747; postmenopausal: OR_≤38 VS >38_ 7.100, 95% CI 3.134–16.084), and represented a better risk predictor among obese women compared to non-obese in pre- and postmenopausal women. Among different molecular subtypes, ORPS was positively correlated with Luminal breast cancer, with additionally positive association with triple-negative breast cancer in premenopausal women. The ORPS might be a potential marker of breast cancer risk among Chinese women.

## Introduction

Breast cancer constitutes the most commonly-diagnosed cancer and the leading cause of cancer death in women, worldwide, according to the International Agency for Research on Cancer (IARC) World Cancer Reports in 2020^1^. Breast cancer was also the first common cancer in Chinese women, with its age-standardized incidence rate increased by 94.73% from 1990 to 2017 and the age-standardized death rate increased by 2.46%^2^. General and central obesity are modifiable risk factors for many chronic diseases^3,4^, and are often defined by body mass index (BMI) or waist-hip ratio (WHR)^5,6^. A meta-analysis of data from 7 cohort studies showed positive associations between BMI and WHR with obesity-related cancers, including postmenopausal breast cancer^7^.

Several interlinked biological pathways have been recognized which may explain the correlation between obesity and breast cancer: (1) alterations in adipocytokine pathophysiology; (2) subclinical chronic low-grade inflammation and oxidative stress; (3) sex hormones bio-synthesis pathway and (4) abnormal system and signaling of insulin-like growth factor (IGF)-1 and insulin resistance^4^. Ten proteins are involved in the former two pathways, namely adiponectin (ADP), leptin (LEP), resistin (RES), visfatin (VF), interleukin-6 (IL-6), tumor necrosis factor-α (TNF-α), C-reactive protein (CRP) and interleukin-8 (IL-8), etc^8^. Nine common circulating sex hormones are involved in the sex hormones biosynthesis pathway, including estrone (E1), estradiol (E2), progesterone (P), estrone sulfate (ES), androstenedione (A), dehydroepiandrosterone (DHA), dehydroepiandrosterone sulfate (DHAS), sex hormone-binding globulin (SHBG) and testosterone (T)^8^. Four proteins including C-peptide, insulin (INS), insulin-like growth factors (IGFs) and IGF-binding proteins (IGFBPs) are taken multiple biological roles in the insulin-IGF-1 axis, one of the potential biological mechanisms underlying the obesity-breast cancer connections^4,8,9^. Abnormal regulations in the blood levels of these proteins caused by adipose tissue may contribute to breast cancer initiation and progression through the activation of multiple signaling pathways^10^.

Although it has been suggested that obesity-related proteins might be suitable for use as markers of breast cancer risk, there are some serious challenges that need to be overcome. For instance, the evidence obtained to date suggests that it is unreliable to use a single obesity-related protein as a risk marker of breast cancer, and it is hard to obtain a comprehensive evaluation especially when the associations between several obesity-related proteins and breast cancer are inconsistent. Using leptin and adiponectin as examples, a meta-analysis of data from 119 studies showed that leptin concentration is positively associated with breast cancer risk, whereas adiponectin is negatively associated^11^. Moreover, the increase in the prevalence of obesity in China has been lower to date than that in Western countries^12^, therefore it is uncertain whether obesity-related proteins might represent equally useful markers of breast cancer risk in Chinese women.

In the present study, we aimed to identify obesity-related proteins that are associated with breast cancer risk using evidence from systematic reviews, and to use this information to establish a robust obesity-related protein score (ORPS) that could be used to assess breast cancer risk.

## Materials and methods

### Study population

An age-matched case–control study was conducted. Two hundred and seventy-nine women newly diagnosed with primary breast cancer by histopathology were enrolled from three hospitals in Chengdu, Sichuan Province, China, from April 2014 to April 2015. The inclusion criteria were Han nationality, living in Sichuan province, not receiving anti-infective treatment, no mental disorder and other malignant tumors. We also excluded patients with metastatic breast cancer. Meanwhile, 260 healthy women of urban and rural screening cohorts from two hospitals in Chengdu, Sichuan Province, China, were recruited from March 2015 to June 2015. The inclusion criteria were Han nationality, living in Sichuan province, and being confirmed to be free of breast cancer via ultrasound or mammography. Women who were receiving anti-infection treatments or had a mental disorder or any malignant tumors were excluded. In both cases and controls, women who had previously undergone abdominoplasty surgery, were receiving neoadjuvant therapy currently or received it in the last 12 months, were receiving or had received any hormonal therapy (HT) during the last 12 months, had gone on a strictly restricted diet in the last 12 months or had lost > 3 kg in the last year were all excluded.

### Information collection

A structured questionnaire was designed based on previous study^13^ to collect information on demographic characteristics and breast cancer risk factors of participants. Women aged 50 or older, or had undergone bilateral oophorectomy or surgical sterilization, or younger than 50 years old but had their menstrual periods been absent for at least 12 months were considered to be postmenopausal. The pathological characteristics of cases, such as the levels of ER (estrogen receptor), PR (progesterone receptor), HER-2 (human epidermal growth factor receptor-2), and Ki-67 (a protein that in humans is encoded by the MKI67 gene) were derived from the hospital information system. The study protocol was approved by the ethics committee of West China School of Public Health and West China Fourth Hospital, Sichuan University. Written informed consents were obtained prior to questionnaires and blood samples donation.

### Selection of detected proteins

Based on the above-mentioned biological pathways^4,14^, electronic databases including PubMed, Embase, CNKI, VIP and Wanfang were assessed to search for original researches or systematic reviews on obesity-related proteins and breast cancer risk. Protocol of this has been registered on the International Prospective Register of Systematic Reviews (PROSPERO no. CRD42019127767). Proteins included in this study should meet the following inclusion criteria: (1) significantly correlated with breast cancer risk; (2) serum or plasma level was relatively stable in vitro with the degradation rate less than 10% or has been detected by previous studies using blood samples stored in the refrigerator for years.

Nine proteins were selected ultimately, namely E2 (estradiol), LEP (leptin), sOB-R (soluble leptin receptor), ADP (adiponectin), RETN (resistin), VF (visfatin), IGF-1 (insulin-like growth factor-1), IGFBP-3 (IGF-binding protein-3) and CRP (C-reactive protein) (Supplementary Table 1). Although E1 is the predominant estrogen in postmenopausal women, E2 was detected in our study because the ability of E2 to act on its receptor is much stronger than that of E1. Additionally, we only detected E2 in postmenopausal women because the majority of E2 in the premenopausal is from the ovary rather than adipose tissue, and its plasma levels are substantially affected by menstrual cycle^15^. Proteins with unclear associations with breast cancer risk were excluded (e.g. C peptide, IL-6, IL-8, etc.). Proteins degrading > 10% in vitro were excluded (e.g. insulin, etc.).

### Blood samples collection and laboratory detection

Whole fasting blood samples were taken in EDTA tubes before physical examination or treatment, and then centrifuged to separate the plasma immediately. Plasma samples were frozen at − 80 ℃ for storage.

Circulating concentrations of the nine obesity-related proteins in plasma were measured by ELISA (Wuhan Elabscience Biotechnology Co., Ltd in China). The standard sample was detected through double-holes and the average value was calculated. Absorbance was detected by the enzyme labeling instrument (Thermo Company, USA). We used Origin software (version 9.0) to obtain the standard curve and calculate the circulating concentrations. To avoid human and instrument errors, each candidate protein was detected by the same researcher using the same batch of ELISA kit.

### Statistical analysis

All analyses were stratified by menopausal status. To avoid the influence of units of proteins, plasma levels were standardized before analyses by dividing the original level by the mean. For continuous variables, if normally distributed, we used mean ± standard deviation (*sd*) and *t* test or variance test to describe and compare the demographic characteristics between cases and controls. Otherwise, median (quartile) and rank-sum test would be used. For categorical variables, we used frequency (composition ratio) and chi-square test. To consider as statistically significant, a *p* < 0.05 is required.

We applied a three-step method to establish the ORPS. First, we constructed a random forest model to obtain the relative weight of each candidate protein. Samples were randomly split into training set and test set by ratios from 50%:50% to 90%:10%^16^. In the training set, ten-fold cross-validation was conducted to identify the best model with the highest AUC among all the split ratios. The final split ratio and its corresponding parameters were determined based on the chosen model. And the importance value of each protein in the random forest model was used as the relative weight coefficient^16^. Finally, using the test set, predictive power of the model was evaluated. Second, two kinds of ORPS were established with linear weighted summation, which were PS_8pre_ or PS_9post_ including eight proteins in premenopausal women or nine proteins in postmenopausal women, and PS_npre_ or PS_npost_ including proteins significantly different between cases and controls in each menopausal status (n was the number of the included proteins). We standardized the scores to 0–100, and the formula for standardization was as follows (1). Third, we did a sensitivity analysis to select the final ORPS from the above two scores. The one with a larger AUC based on the Delong Test was chosen. The ROC curve was drawn to obtain the cut-off value of the chosen score where the Youden index was the highest.

The associations between the final ORPS and breast cancer risk in different menopausal status and WHR level were analyzed using Logistic regression. Subgroup analysis stratified by BMI was not conducted, because BMI has been shown to be less accurate for assessing obesity in certain groups of people including Asians. Central obesity defined by WHR ≥ 0.85 was defined as the appropriate measurement of obesity in our study^17^.

1 $${\text{Standardized score}} = 100*{\frac{{{\text{The original score}} - {\text{Min}}_{{\text{the original score}}} }}{{{\text{Max}}_{{\text{the original score}}} - {\text{Min}}_{{\text{the original score}}}} }}$$

All the statistical analyses were conducted using SPSS 24 software (IBM Corp, Armonk, NY, USA. https://www.ibm.com/support/knowledgecenter/zh/SSLVMB_24.0.0/spss/product_landing.html), R 3.5.1 software (R Core Team (2018), Vienna, Austria. https://www.R-project.org/) and Microsoft EXCEL software (Microsoft (2016), USA. https://www.microsoft.com/zh-cn/microsoft-365/excel). An overview of the analysis process was shown in Supplementary Fig. 1.

### Ethics approval

The study protocol was approved by the ethics committee of West China School of Public Health and West China Fourth Hospital, Sichuan University. All subjects participated in the study voluntarily and signed informed consent forms.

### Informed consent

Informed consent was obtained from all individual participants included in this study.

## Results

### Demographic characteristics of participants

167 premenopausal cases and 149 premenopausal controls with the same median age of 44 years old were enrolled. Meanwhile, 112 postmenopausal cases with a median age of 57.5 years old and 111 postmenopausal controls with the median age of 56 years old were included. For both pre- and postmenopausal cases, they had more energy intake daily, lower WHR, more adverse history of pregnancy, more abortion, lower cultural level, lower income, different occupation and place of residence compared to controls (cases had lower levels of WHR but higher levels of energy intake because obesity is also related to other factors such as genetic background, basic metabolism and physical activity). Additionally, premenopausal cases had an earlier age at menarche and exercised less, and postmenopausal cases were more likely to be passive smokers and have more estrogen diseases (Supplementary Table 2). Significantly higher levels of RETN and CRP and lower levels of sOB-R and ADP existed in cases irrespective of menopausal status, while lower levels of IGFBP-3 existed only in premenopausal cases (Table 1 and Supplementary Fig. 2).

**Table 1:** The distribution and comparison of proteins among controls and cases of different menopausal status.

	Premenopausal			Postmenopausal		
	Cases (n = 167)	Controls (n = 149)	z/x^2^ (*p* values)	Cases (n = 112)	Controls (n = 111)	z/x^2^ (*p* values)
E2 (pg/mL)	Not detected	Not detected	–	10.95 (8.12, 13.15)	10.09 (6.40, 12.88)	− 1.35 (0.177)
RETN (μg/L)	20.00 (9.69, 40.42)	12.55 (4.97, 24.52)	− 3.74 (< 0.001)*	53.21 (31.55, 105.81)	44.10 (24.45, 71.45)	− 2.09 (0.037)*
VF (μg/L)	6.76 (3.59, 11.70)	7.72 (4.35, 13.00)	− 1.27 (0.205)	5.34 (2.90, 12.82)	6.77 (3.47, 15.02)	− 1.06 (0.290)
LEP (μg/L)	8.34 (4.00, 14.11)	8.82 (4.63, 14.18)	− 0.78 (0.437)	7.98 (4.71, 12.54)	8.22 (4.38, 13.76)	− 0.06 (0.956)
sOB-R (ng/mL)	24.55 (13.15, 38.80)	30.10 (19.44, 44.03)	− 2.55 (0.011)*	22.49 (13.31, 35.48)	31.57 (18.40, 41.80)	− 2.39 (0.017)*
ADP (µg/mL)	10.68 (7.21, 14.90)	13.83 (8.98, 17.69)	− 3.69 (< 0.001)*	12.01 (9.18, 16.63)	19.57 (10.07, 27.57)	− 4.71 (< 0.001)*
CRP (mg/L)	2.51 (0.83, 7.02)	1.45 (0.61, 4.10)	− 2.38 (0.018)*	4.32 (1.71, 10.33)	1.98 (0.77, 5.93)	− 3.08 (0.002)*
IGF-1 (ng/mL)	86.92 (60.67, 171.77)	99.51 (47.58, 207.76)	− 0.15 (0.885)	89.60 (60.56, 196.18)	128.91 (61.61, 257.98)	− 1.28 (0.200)
IGFBP-3 (ng/mL)	277.45 (161.77, 481.44)	355.15 (227.93, 515.91)	− 2.42 (0.016)*	316.88 (184.66, 467.36)	330.55 (232.19, 505.60)	− 1.42 (0.157)

Described as the median (25%, 75%) and rank sum test was used.

*E2* estradiol, *LEP* leptin, *sOB-R* soluble leptin receptor, *ADP* adiponectin, *RETN* resistin, *VF* visfatin, *IGF-1* insulin-like growth factor 1, *IGFBP-3* insulin-like growth factor binding protein 3, *CRP* C-reactive protein.

*p < 0.05.

### The weight coefficients of proteins

The samples were split into train set and test set according to 80%:20% for premenopausal women with the largest AUC of 0.680 based on the train set. Among all the proteins, the order of the weight coefficients from the highest to the lowest were RETN, ADP, IGF-1, IGFBP-3, CRP, sOB-R, LEP and VF. (Supplementary Table 3). For postmenopausal women, they were split into train set and test set according to 60%:40% with the largest AUC of 0.798 for the train set. The order of the weight coefficients from the highest to the lowest were ADP, RETN, IGF-1, CRP, E2, VF, IGFBP-3, sOB-R, and LEP (Supplementary Table 3). The weight coefficient of LEP was the last in postmenopausal women and the penultimate in premenopausal women.

### The distribution of ORPS

**Figure 1:**
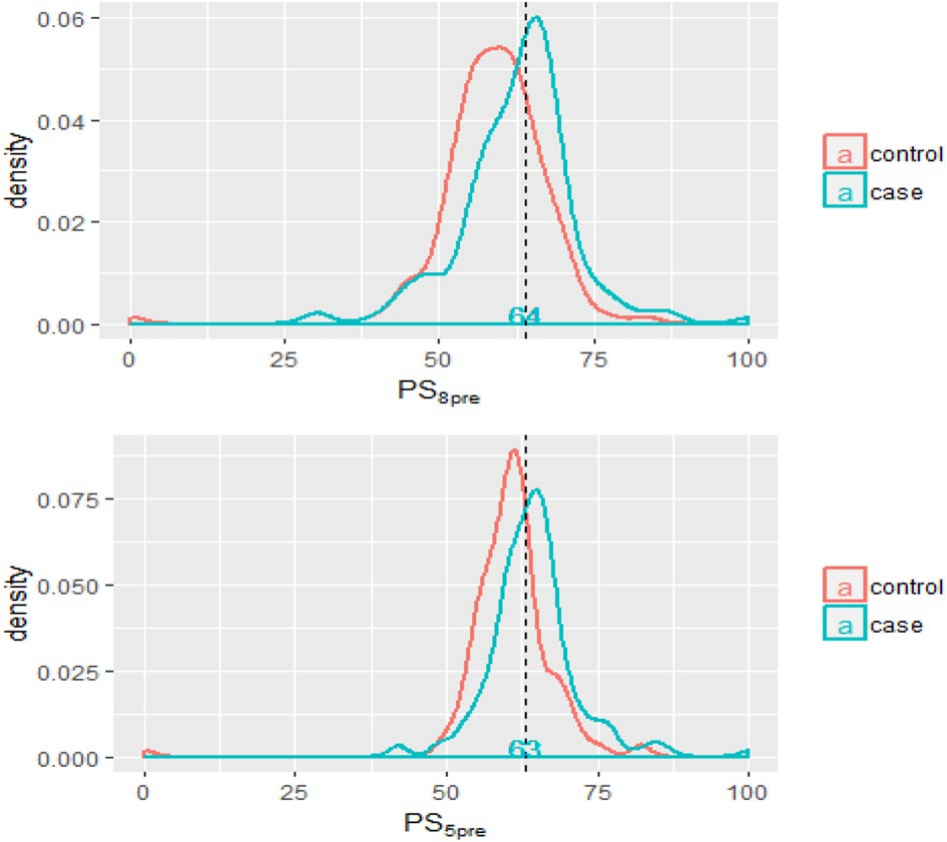
The distribution curve of PS_8pre_ and PS_5pre_ among controls and cases of premenopausal women (the dashed line was the cut-off value based on ROC). The curve was conducted using R 3.5.1 software (R Core Team, Vienna, Austria, 2018. https://www.R-project.org/) and Microsoft EXCEL software (Microsoft, USA, 2016. https://www.microsoft.com/zh-cn/microsoft-365/excel).

**Figure 2:**
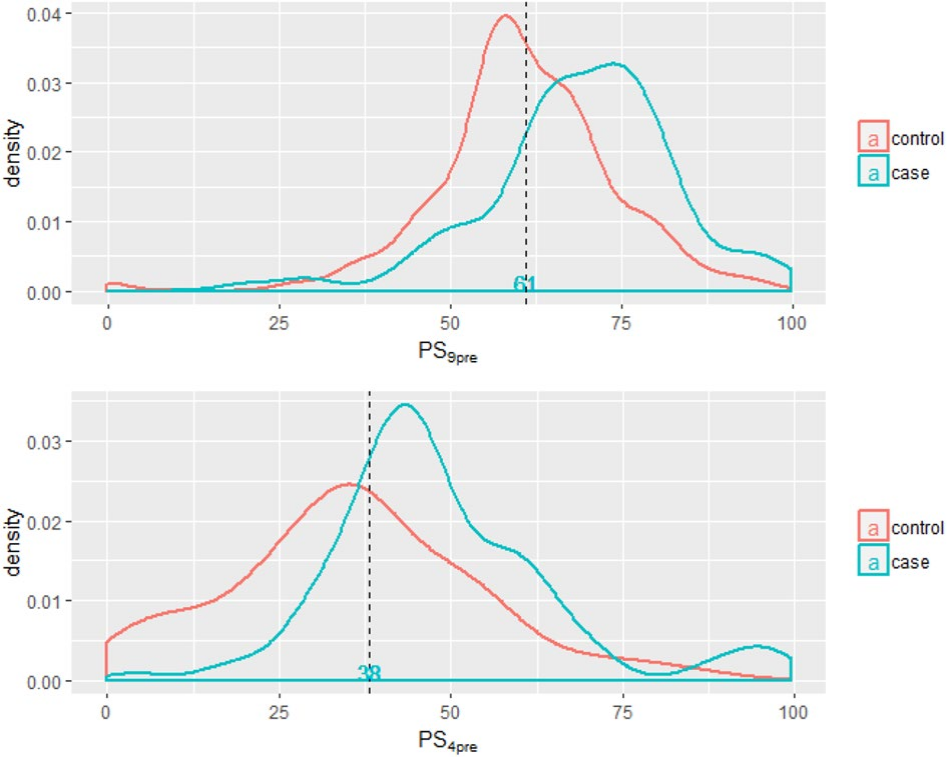
The distribution curve of PS_9post_ and PS_4post_ among controls and cases of postmenopausal women (the dashed line was the cut-off value based on ROC). The curve was conducted using R 3.5.1 software (R Core Team, Vienna, Austria, 2018. https://www.R-project.org/) and Microsoft EXCEL software (Microsoft, USA, 2016. https://www.microsoft.com/zh-cn/microsoft-365/excel).

The formula of ORPS was shown in Eqs. (2–5). The range of PS_8pre_ of premenopausal controls was 0–84 with a median of 60 while the range of PS_5pre_ was 0–82 with a median of 61. For premenopausal cases, PS_8pre_ was scored in the range of 30–100 with a median of 64, and PS_5pre_ was scored in the range of 42–100 with a median of 64 (Fig. 1). Among postmenopausal women, the range of PS_9post_ in the controls was 0–95 with a median of 60 while the PS_4post_ was scored in the range of 0–88 with a median of 36. For cases, the PS_9post_ was scored in the range of 20–100 with a median of 71 while the range of PS_4post_ was 4–100 with a median of 46 (Fig. 2).

2 $$\begin{aligned} {\text{PS}}_{{{\text{8pre}}}} & = ({19}.{1}0{6}0{9}*{\text{RETN}}_{{\text{s}}} ) + \left( {{14}.{761}0{3}*{\text{CRP}}_{{\text{s}}} } \right) - \left( {{18}.00{972}*{\text{ADP}}_{{\text{s}}} } \right) - \left( {{16}.{44989}*{\text{IGFBP}} - {3}_{{\text{s}}} } \right) \\ & \quad - \left( {{14}.{3512}*{\text{sOB}} - {\text{R}}_{{\text{s}}} } \right) - \left( {{16}.{79}0{15}*{\text{IGF}} - {1}_{{\text{s}}} } \right) - \left( {{12}.{77187}*{\text{VF}}_{{\text{s}}} } \right) - \left( {{13}.{86147}*{\text{LEP}}_{{\text{s}}} } \right) \\ \end{aligned}$$

3 $$\begin{aligned} {\text{PS}}_{{{\text{5pre}}}} & = ({19}.{1}0{6}0{9}*{\text{RETN}}_{{\text{s}}} ) + \left( {{14}.{761}0{3}*{\text{CRP}}_{{\text{s}}} } \right) - \left( {{18}.00{972}*{\text{ADP}}_{{\text{s}}} } \right) \\ & \quad - \left( {{16}.{44989}*{\text{IGFBP}} - {3}_{{\text{s}}} } \right) - \left( {{14}.{3512}*{\text{sOB}} - {\text{R}}_{{\text{s}}} } \right) \\ \end{aligned}$$

4 $$\begin{aligned} {\text{PS}}_{{{\text{9post}}}} & = \left( {{6}.{169918}*{\text{E2}}_{{\text{s}}} } \right) + \left( {{7}.{788563}*{\text{RETN}}_{{\text{s}}} } \right) + \left( {{6}.{191498}*{\text{CRP}}_{{\text{s}}} } \right) - \left( {{18}.{441782}*{\text{ADP}}_{{\text{s}}} } \right) \\ & \quad - \left( {{5}.{688581}*{\text{sOB}} - {\text{R}}_{{\text{s}}} } \right) - \left( {{5}.{963165}*{\text{IGFBP}} - {3}_{{\text{s}}} } \right) - \left( {{6}.{8}0{4443}*{\text{IGF}} - {1}_{{\text{s}}} } \right) - \left( {{6}.0{74258}*{\text{VF}}_{{\text{s}}} } \right) - \left( {{3}.{9281}0{3}*{\text{LEP}}_{{\text{s}}} } \right) \\ \end{aligned}$$

5 $${\text{PS}}_{{{\text{4post}}}} = \left( {{7}.{788563}*{\text{RETN}}_{{\text{s}}} } \right) + \left( {{6}.{191498}*{\text{CRP}}_{{\text{s}}} } \right) - \left( {{18}.{441782}*{\text{ADP}}_{{\text{s}}} } \right) - \left( {{5}.{688581}*{\text{sOB}} - {\text{R}}_{{\text{s}}} } \right)$$

### Association of ORPS and breast cancer risk

**Figure 3:**
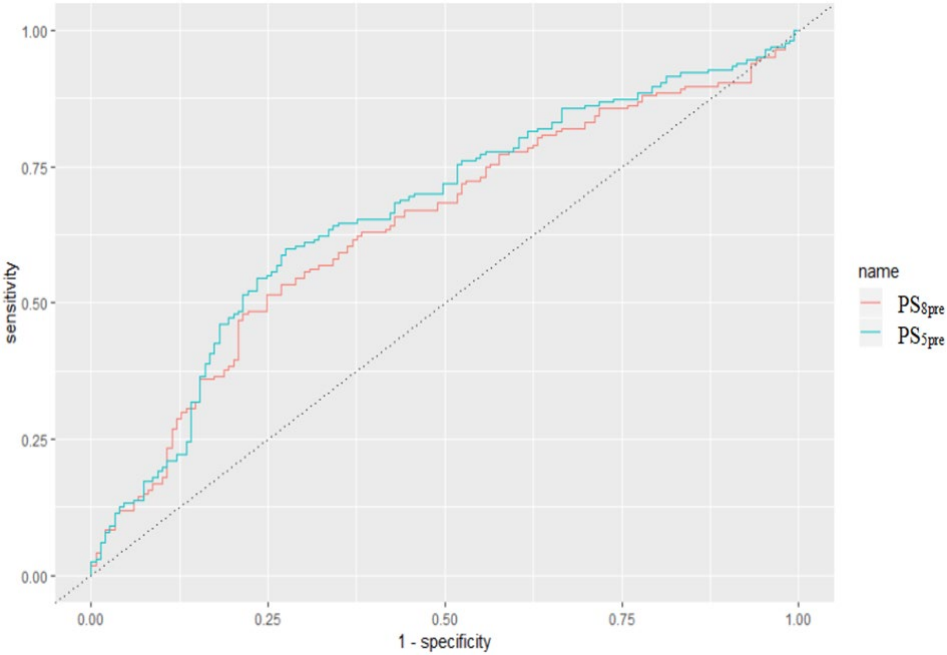
The ROC of PS_8pre_ and PS_5pre_ among premenopausal women. The AUC of PS_8pre_ and PS_5pre_ were 0.641 and 0.666 (*p* = 0.23). The curve was conducted using R 3.5.1 software (R Core Team, Vienna, Austria, 2018. https://www.R-project.org/) and Microsoft EXCEL software (Microsoft, USA, 2016. https://www.microsoft.com/zh-cn/microsoft-365/excel).

**Figure 4:**
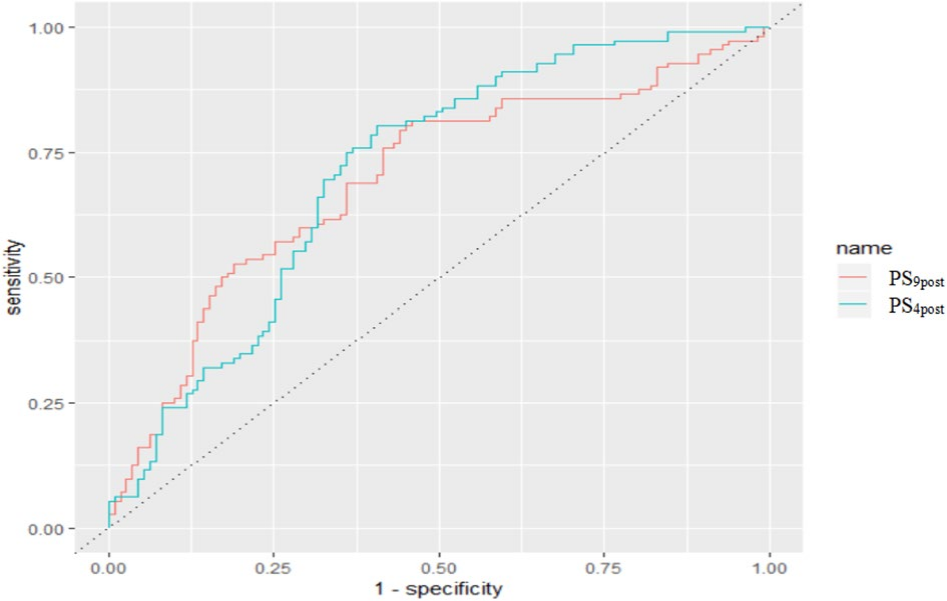
The ROC of PS9post and PS4post among postmenopausal women Note: The AUC of PS9post and PS4post was 0.696 and 0.712 (p = 0.52). The curve was conducted using R 3.5.1 software (R Core Team, Vienna, Austria, 2018. https://www.R-project.org/) and Microsoft EXCEL software (Microsoft, USA, 2016. https://www.microsoft.com/zh-cn/microsoft-365/excel).

For premenopausal women, PS_5pre_ was chosen as the final score with the same AUC (0.666 VS 0.641, *p* = 0.23) and fewer proteins compared to that of PS_8pre_ (Fig. 3). The cut-off value based on the ROC of PS_5pre_ was 63 with the highest Yoden Index of 0.324. PS_5pre_ was composed of 5 proteins, including RETN, ADP, sOB-R, CRP, and IGFBP-3. For postmenopausal women, PS_4post_ was chosen as the final score with the same AUC and fewer proteins than that of PS_9post_ (0.712 VS 0.696, *p* = 0.52) (Fig. 4). The cut-off value divided by the ROC of PS_4post_ was 38 with the highest Yoden Index of 0.399. It was composed of RETN, ADP, sOB-R and CRP.

**Figure 5:**
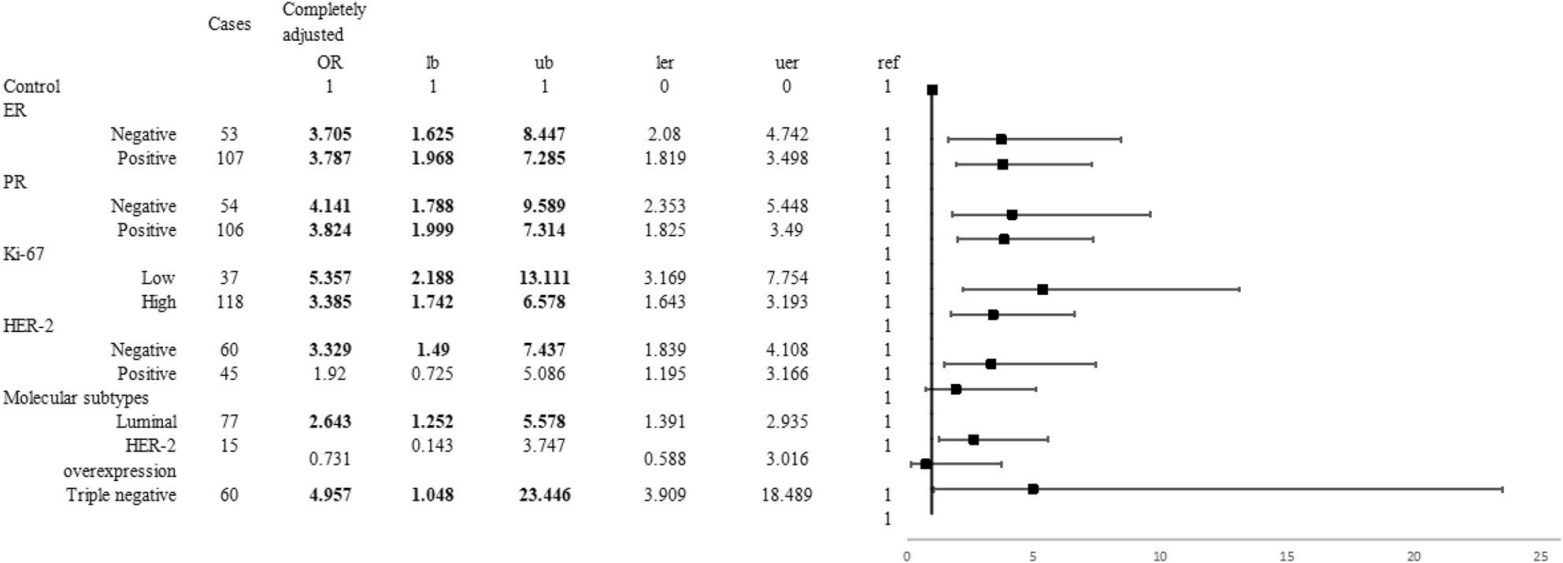
The association between PS_5pre_ and the risk of premenopausal breast cancer risk of different molecular subtypes. *lb the lower limit of confidence interval, *ub* the upper limit of confidence interval, *ler* the distance from OR to the lower limit of confidence interval, *uer* the distance from OR to the upper limit of confidence interval, *ref* OR = 1. The figures were conducted using R 3.5.1 software (R Core Team, Vienna, Austria, 2018. https://www.R-project.org/) and Microsoft EXCEL software (Microsoft, USA, 2016. https://www.microsoft.com/zh-cn/microsoft-365/excel).

**Figure 6:**
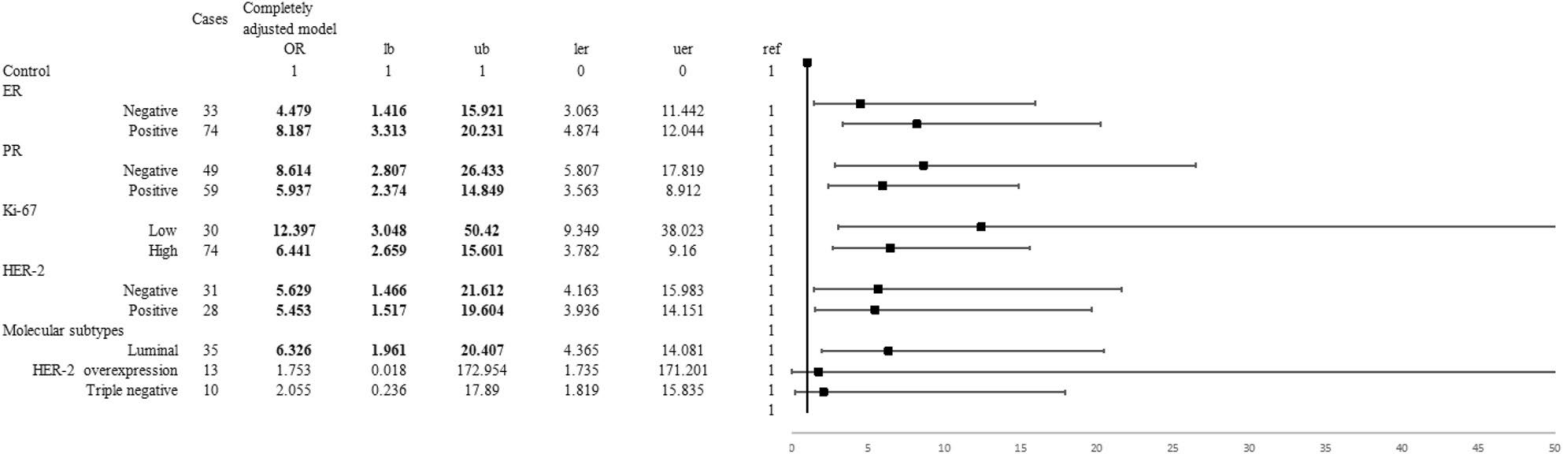
The association between PS_4post_ and the risk of postmenopausal breast cancer risk of different molecular subtypes. *lb the lower limit of confidence interval, *ub* the upper limit of confidence interval, *ler* the distance from OR to the lower limit of confidence interval, *uer* the distance from OR to the upper limit of confidence interval, *ref* OR = 1. The figures were conducted using R 3.5.1 software (R Core Team, Vienna, Austria, 2018. https://www.R-project.org/) and Microsoft EXCEL software (Microsoft, USA, 2016. https://www.microsoft.com/zh-cn/microsoft-365/excel).

When common risk factors were adjusted, PS_5pre_ was positively correlated with premenopausal breast cancer risk (OR_≤63vs>63_ 3.696, 95% CI 2.025–6.747) (Table 2). And PS_5pre_ was positively correlated with Luminal and triple-negative breast cancer (OR_Luminal_ 2.643, 95% CI 1.252–5.578; OR_triple-negative_ 4.957, 95% CI 1.048–23.446) (Fig. 5). Meanwhile, PS_4post_ was positively correlated with postmenopausal breast cancer risk (OR_≤38vs>38_ 7.100, 95% CI 3.134–16.084) (Table 3). And among several breast cancer subtypes, only Luminal breast cancer risk was positively correlated with PS_4post_ (OR_Luminal_ 6.326, 95% CI 1.961–20.407) (Fig. 6).

**Table 2:** The association between PS8pre or PS5pre and the risk of premenopausal breast cancer.

	Cases (n = 167)	Controls (n = 149)	Crude model^b^		Adjusted model^b^		Completely adjusted model^c^	
			OR (95% CI)	AUC	OR (95% CI)	AUC	OR (95% CI)	AUC
PS_8pre_, 50% (25%, 75%)	64 (58, 68)	60 (55, 64)	1.051 (1.022, 1.081)	0.641	1.043 (1.009, 1.077)	0.831	1.040 (1.007, 1.075)	0.841
PS_5pre_, 50% (25%, 75%)	64 (60, 67)	61 (57, 63)	1.083 (1.041, 1.126)	0.666	1.076 (1.029, 1.125)	0.835	1.070 (1.023, 1.119)	0.846
**Quartiles of PS**_**5pre**_^**a**^**, n (%)**								
[0, 57]	19 (11.4%)	34 (22.8%)	1.00	0.664	1.00	0.849	1.00	0.854
(57, 61]	31 (18.6%)	43 (28.9%)	1.290 (0.624, 2.669)		1.011 (0.408, 2.502)		1.009 (0.404, 2.525)	
(61, 63]	17 (10.2%)	29 (19.5%)	1.049 (0.462, 2.383)		0.952 (0.347, 2.613)		0.902 (0.324, 2.508)	
(63, 100]	100 (59.9%)	43 (28.9%)	4.162 (2.139, 8.096)		4.053 (1.768, 9.289)		3.606 (1.562, 8.325)	
			***p***_**trend**_ < 0.001		***p***_**trend**_ < 0.001		***p***_**trend**_ < 0.001	
**Cut-off value of PS**_**5pre**_**, n (%)**								
[0, 63]	67 (40.1%)	106 (71.1%)	1.00	0.655	1.00	0.849	1.00	0.854
(63, 100]	100 (59.9%)	43 (28.9%)	3.679 (2.299, 5.889)		4.087 (2.257, 7.402)		3.696 (2.025, 6.747)	

^a^Defined by the quartile level in the control group.

^b^Crude model: Only the score, without other environmental risk factors. Adjusted model: Adjusting age at menarche, residence, education, income, occupation, adverse pregnancy history, abortion history, energy intake and exercise.

^c^Completely adjusted model: Adjusting WHR further. The sensitivity was 0.70, specificity was 0.52, positive predictive value was 0.62, negative predictive value was 0.61 and accuracy was 0.62 when only PS_5pre_ was included. According to the results of analysis, the number of proteins significantly different between groups was 5 of the premenopausal subjects, so the two scores were recorded as PS_8pre_ and PS_5pre_ respectively.

**Table 3:** The association between PS9post or PS4post and the risk of postmenopausal breast cancer.

	Cases (n = 112)	Controls (n = 111)	Crude model^b^		Adjusted model^b^		Completely adjusted model^c^	
			OR (95%CI)	AUC	OR (95%CI)	AUC	OR (95%CI)	AUC
PS_9post_, 50% (25%, 75%)	71 (63, 78)	60 (55, 69)	1.050 (1.026, 1.074)	0.696	1.052 (1.023, 1.081)	0.851	1.053 (1.023, 1.083)	0.865
PS_4post_, 50% (25%, 75%)	46 (39, 56)	36 ( (25, 48)	1.046 (1.027, 1.066)	0.712	1.046 (1.022, 1.070)	0.856	1.046 (1.021, 1.071)	0.867
**Quartiles of PS**_**4post**_^**a**^**, n (%)**								
[0, 25]	4 (3.6%)	29 (26.1%)	1.000	0.683	1.000	0.851	1.000	0.867
(25, 37]	14 (12.5%)	26 (23.4%)	3.904 (1.140, 13.367)		3.100 (0.690, 13.935)		3.135 (0.666, 14.745)	
(37, 49]	50 (44.6%)	29 (26.1%)	12.500 (3.993, 39.128)		8.720 (2.144, 35.464)		8.970 (2.118, 37.984)	
(49, 100]	44 (39.3%)	27 (24.3%)	11.815 (3.741, 37.312)		9.043 (2.188, 37.365)		9.373 (2.175, 40.390)	
			*p*_trend_ < 0.001		p_trend_ < 0.001		*p*_trend_ < 0.001	
**Cut-off value of PS**_**4post**_**, n (%)**								
[0, 38]	22 (19.6%)	66 (59.5%)	1.000	0.699	1.000	0.868	1.000	0.881
(38, 100]	90 (80.4%)	45 (40.5%)	6.000 (3.291, 10.941)		6.671 (3.041, 14.637)		7.100 (3.134, 16.084)	

^a^Defined by the quartile level in the control group.

^b^Crude model: Only the score, without other environmental risk factors. Adjusted model: Adjusting residence, education, income, occupation, passtive smoking, adverse pregnancy history, abortion history, energy intake and estrogen disease.

^c^Completely adjusted model: Adjusting WHR further. The sensitivity was 0.65, specificity was 0.68, positive predictive value was 0.68, negative predictive value was 0.66 and accuracy was 0.67 when only PS_4post_ was included. The number of proteins significantly different between groups was 4 of the postmenopausal subjects, so the two scores were recorded as PS_9post_ and PS_4post_ respectively.

### ORPS and breast cancer risk in different WHR levels

With regard to subgroup analysis stratified by WHR levels, PS_5pre_ was positively relevant to premenopausal breast cancer risk in all WHR levels, with higher OR in obese women (WHR > 0.85) (OR_WHR≤0.85_ 3.918, 95% CI 1.280–11.996; OR_WHR>0.85_ 6.941, 95% CI 2.649–18.185) (Table 4). The association of PS_4post_ with postmenopausal breast cancer risk in WHR ≤ 0.85 was indistinguishable temporarily owing to a small sample size (OR_WHR≤0.85_ NA). However, PS_4post_ was positively correlated with the risk in obese women (OR_WHR>0.85_ 4.984, 95% CI 1.883–13.190) (Table 4).

**Table 4:** The association between PS_5pre_, PS_4post_ and the risk in different WHR.

	WHR ≤ 0.85				WHR > 0.85			
	Cases (n, %)	Controls (n, %)	Crude model^a^ (OR, 95% CI)	Adjusted model^b^ (OR, 95% CI)	Cases (n, %)	Controls (n, %)	Crude model^a^ (OR, 95% CI)	Adjusted model^b^ (OR, 95% CI)
**Premenopausal**								
PS_5pre_								
[0, 63]	29 (35.4%)	27 (58.7%)	2.597 (1.238, 5.450)	3.918 (1.280, 11.996)	38 (44.7%)	79 (76.7%)	4.071 (2.178, 7.612)	6.941 (2.649, 18.185)
(63, 100]	53 (64.6%)	19 (41.3)			47 (55.3%)	24 (23.3%)		
**Postmenopausal**								
PS_4post_								
[0, 38]	10 (23.3%)	14 (70.0%)	7.700 (2.343, 25.301)	NA	12 (17.4%)	52 (57.8%)	6.50 (3.070, 13.760)	4.984 (1.883, 13.190)
(38, 100]	33 (76.7%)	6 (30.0%)			57 (82.6%)	38 (42.2%)		

^a^Crude model: only the score, without other environmental risk factors.

^b^Adjusting age at menarche, residence, education, income, occupation, adverse pregnancy history, abortion history, energy intake and exercise in adjusted model of PS_5pre._ Adjusting residence, education, income, occupation, passive smoking, adverse pregnancy history, abortion history, energy intake and estrogen disease in adjusted model of PS_4post._

## Discussion

Based on evidence from systematic reviews and population studies, we selected nine candidate proteins to calculate the obesity-related protein scores (ORPS). As a result, the PS_5pre_ or PS_4post_, which was positively correlated with breast cancer risk, shared the same AUC as that of the PS_8pre_ or PS_9post_, but with fewer proteins included. Both PS_5pre_ and PS_4post_ were composed of RETN, ADP, CRP and sOB-R with an additional IGFBP-3 for PS_5pre_, and these five proteins were all significantly different between controls and cases. We found higher levels of RETN and CRP and lower levels of ADP and sOB-R in cases in both menopausal status, and lower levels of IGFBP-3 in only premenopausal cases. Additionally, ORPS seems to represent a better risk predictor among obese women compared to non-obese in pre- and postmenopausal women.

RETN concentration is associated with obesity and insulin resistance^18^. It could inhibit the hypoglycemic reactivity and sensitivity of insulin, resulting in hyperinsulinemia and other chronic diseases^19,20^. RETN is also involved in tumor progression through the activation of inflammation and the expression of adhesion molecules, promoting the proliferation, metabolism and invasion of tumor cells^21,22^. A meta-analysis of data from eight previous studies found that RETN concentration positively correlated with breast cancer risk^23^, which is consistent with our findings.

CRP is one kind of acute-phase response proteins related to obesity^24^ and chronic inflammation. Previous studies have suggested that chronic inflammation often coexists in tissues that are affected by malignant tumors. Besides, cancer cells could aggravate chronic inflammation response and impart tumor micro-environment, contributing to the proliferation and survival of malignant cells, angiogenesis and metastasis, destruction of adaptive immunity, and decreasing responses to hormones and chemotherapy^25–27^. Similar to the present findings, a systematic review of 15 cohort and case–control studies suggested a positive association between CRP and breast cancer risk^28^.

ADP is negatively correlated with obesity^18^ and risk of breast cancer by activating an anti-proliferative process on cancer cells, controlling cell cycle, regulating cell migration and inhibiting DNA synthesis, as well as anti-tumor immunity^29,30^. The protective effect on breast cancer, that in line with our study, was also observed in a systematic review consisting of 60 observational studies^31^.

sOB-R binds another adipocytokine, namely LEP. LEP is mainly involved in obesity, appetite control, energy metabolism^18^ and tumor progression by inhibiting the apoptosis of tumor cells and promoting the expression of oncogenes. Circulating sOB-R binds LEP, which results in a low concentration of free LEP and the inhibition of its biological function^22,32^. A previous case–control study found that the sOB-R concentration in normal individuals was higher than that in breast cancer cases^33^.

IGF-1 may contribute to the development of obesity^34^. And it promotes mitosis, proliferation, and differentiation, and inhibits apoptosis, but these are inhibited when IGF-1 binds to IGFBP-3, one of its specific binding proteins^35,36^. The carcinogenic mechanism of IGFBP-3 was suggested to be bidirectional^37^. A systematic review showed that high levels of IGF-1 and IGFBP-3 were positively correlated with premenopausal breast cancer risk^38^, while IGFBP-3 was found to have a protective effect in our study, which might be due to the bidirectional carcinogenic mechanism of IGFBP-3. Additionally, the above association was found only in premenopausal women. The possible explanation is: since each of these molecules may be regulated by estrogen, which may increase membrane IGF-1 receptors, which would increase the effects of IGF-1, and downregulate IGFBP-3, such that the high estrogen concentrations in premenopausal women would amplify the biological effect of IGF-1^35^.

Additionally, we also found that PS_5pre_ and PS_4post_ was positively associated with Luminal breast cancer risk, which may be related to the potential interactions among proteins and hormone receptors. In vitro, the pro-apoptotic effect of ADP was found only in ER+ cell lines^39^. The signal axis of leptin interacts with ER to enhance the occurrence and metastasis of tumors^40^. In addition, we have shown that there are lower ADP concentrations in Luminal breast cancer^41^. Cho et al*.*^42^ suggested that a higher concentration of LEP results in a poorer prognosis in ER or PR-positive patients only, whereas Lee et al*.*^43^ found that ER-positive patients have a higher concentration of RETN than ER-negative patients. Furthermore, Raman et al*.*^44^ showed that high CRP concentrations are associated with PR or ER. Therefore, the proteins that comprised PS_5pre_ and PS_4post_ all interacted with ER or PR.

Moreover, it was interesting to note that the PS_5pre_ had shown a stronger relevance to triple-negative breast cancer than that of luminal breast cancer in premenopausal women. The association between PS_5pre_ and triple-negative breast cancer could be mediated via IGFBP-3 by two ways. First, IGFBP-3 and IGF-1 are regulated by estrogen^35^, resulting in low concentrations of IGFBP-3 in premenopausal women, which is consistent with our finding. Second, via the epidermal growth factor receptor signaling pathway^45^, the over-expressed IGFBP-3 may promote triple-negative breast cancer development^46,47^. Therefore, in ER-negative breast cancer, high concentrations of IGFBP-3 would result in more rapid recurrence, and therefore a poorer prognosis^48^. Thus, IGFBP-3 may represent a novel predictor of and potential therapeutic target for triple-negative premenopuasal breast cancer.

This study is the first to construct the ORPS for breast cancer. We selected our candidate proteins based on high-quality evidence, and constructed the ORPS with an innovative three-step method. Our study also had several weaknesses. As a case–control study, the association between ORPS and breast cancer risk could not been confirmed. The applicability and authenticity need to be further verified by future longitudinal studies. Additionally, due to a small sample size, the statistical power might be low and the associations might not be estimated correctly, and we did not further subdivide Luminal breast cancers into Luminal-A and Luminal-B subtypes. Finally, complex interactions between the investigated proteins should be further considered. Despite all these, the associations of proteins and breast cancer risk we observed were consistent with the previous studies, and a significant association was found between the ORPS and breast cancer risk.

## Conclusion

ORPS (PS_5pre_ or PS_4post_) was positively correlated with breast cancer risk and represented a better risk predictor among obese women compared to non-obese in pre- and postmenopausal women. Among different molecular subtypes, ORPS was positively correlated with Luminal breast cancer with additionally positive association with triple-negative breast cancer in premenopausal women. The ORPS might be a potential marker of breast cancer risk among Chinese women. Future studies should focused on the development of rapid detection methods of obesity-related proteins and some of their components in isolation, so as to promote the application of ORPS in clinical practice.

## Supplementary Information

Supplementary Information.

## Data Availability

Contact the corresponding author if necessary.
