# Supplementary material for: Obesity-related proteins score as a potential marker of breast cancer risk
Source: Sci Rep. 2021 Apr 15;11:8230. doi: 10.1038/s41598-021-87583-3 (PMC8050206; doi:10.1038/s41598-021-87583-3)
Supplement: Supplementary file 1 — Supplementary Information. [file 41598_2021_87583_MOESM1_ESM.pdf]

Obesity-related proteins score as a potential marker of breast cancer risk

Sha Diao[1,2a], Xueyao Wu[1b], Xiaofan Zhang[1], Yu Hao[1], Bin Xu[1], Xu Li[1,3], Lulu Tian[1], Yunqi Miao[1], Xunying Zhao[1], Feng Ye PhD[4*], Jiayuan Li PhD[1*]

[a,b] The first two authors contributed equally to this work.

[1]Department of Epidemiology and Health Statistics, West China School of Public Health and West China Fourth Hospital, Sichuan University, Chengdu 610041, Sichuan, China; [2]Department of Pharmacy, West China Second University Hospital, Sichuan University, Chengdu 610041, Sichuan, China; [3]Department of Clinical Research Management, West China Hospital, Sichuan University, Chengdu 610041, Sichuan, China; [4]Institute of Clinical Pathology, West China Hospital, Sichuan University, Chengdu 610041, Sichuan, China

***Corresponding Author**

Jiayuan Li, PhD

Department of Epidemiology and Health Statistics, West China School of Public Health and West China Fourth Hospital, Sichuan University, Chengdu 610041, Sichuan, China.

Email: lijiayuan73@163.com

Feng Ye PhD

Institute of Clinical Pathology, West China Hospital, Sichuan University, Chengdu 610041, Sichuan, China

Email: University.fengye@scu.edu.cn

Supplementary Table 1 List of proteins in four pathways positively associated with the risk of breast cancer in population-based studies

		Population-based studies			Feasibility				Selected or not	
Pathways	Proteins	Association	Study type	Levels of Evidence*	Detection method	Sample	Volume	Stability in vitro	Selected in our study	Reasons for not selected
Estrogen pathway	E2	Premenopausal: Weak positive correlation	Meta analysis of 7 prospective studies [1]	2a	Radioimmunity or mass spectrometry or ELISA	Serum or plasma	50.0-100.0ul	Yes[2, 3]	Yes, but only for postmenopausal female	E2 is secreted by the ovary instead of obese tissue for premenopausal female, and the level of E2 is affected by menstrual cycle greatly.
		Postmenopausal: Positive	Reanalysis of 9 prospective studies[4]	2a	Radioimmunity or mass spectrometry or ELLISA	Serum or plasma	50.0-100.0ul			
Adipocytokine pathway	LEP	Positive	Meta analysis of 35 observational studies[5]	3a	ELISA	Serum or plasma	10.0ul (based on preliminary experiments)	Yes[6]	Yes	-
	sOB-R	Negative	Single observational studies[7, 8]	3b	ELISA	Serum or plasma	24.0ul (based on preliminary experiments)	Yes&[9]	Yes	-
	ADP	Negative	Meta analysis of 31 observational studies[10]	3a	ELISA	Serum or plasma	0.1ul (based on preliminary experiments)	Yes&[11]	Yes	-
	RETN	Positive	Meta analysis of 6 observational studies[12]	3a	ELISA	Serum or plasma	1.5ul (based on preliminary experiments)	Yes&[13]	Yes	-
	VF	Positive	Meta analysis of 3	3a	ELISA	Serum or	15.0ul (based on	Yes&[11]	Yes	-

Supplementary Table 1 List of proteins in four pathways positively associated with the risk of breast cancer in population-based studies

		Population-based studies			Feasibility				Selected or not	
Pathways	Proteins	Association	Study type	Levels of Evidence*	Detection method	Sample	Volume	Stability in vitro	Selected in our study	Reasons for not selected
			observational studies[12]			plasma	preliminary experiments)			
Insulin pathway	IGF-1	Positive	Pooled analysis of 17 prospective studies[14]	2a	ELISA	Serum or plasma	24.0μl	Yes[6]	Yes	-
	IGFBP-3	Weak positive correlation	Pooled analysis of 17 prospective studies[14]	2a	ELISA	Serum or plasma	1.2μl	Yes[6]	Yes	-
	C peptide	Unclear	Meta analysis of 12 observational studies[15]	3a	ELISA	Serum or plasma	120.0μl	Yes[6]	No	The association is unclear, and it requires a large amount of plasma/serum to detect.
	Insulin	Unclear	Meta analysis of 12 observational studies[15]	3a	ELISA	Serum or plasma	24.0μl	No（based on preliminary experiments）	No	The association is unclear, and it is not stable in vitro.
Inflammatory pathways	CRP	Positive	Meta analysis of 12 cohort studies[16]	2a	ELISA	Serum or plasma	1.0μl	Yes[17]	Yes	-
	IL-6	Unclear(Positive correlation based on Meta analysis	Meta analysis of 29 observational studies[12]+3	3a	ELISA	Serum or plasma	120.0μl	Yes[17, 21]	No	The mechanism between cancer and inflammation is bidirectional. However, the results of prospective

Supplementary Table 1 List of proteins in four pathways positively associated with the risk of breast cancer in population-based studies

		Population-based studies			Feasibility				Selected or not	
Pathways	Proteins	Association	Study type	Levels of Evidence*	Detection method	Sample	Volume	Stability in vitro	Selected in our study	Reasons for not selected
		irrespective of study types; Irrelevant based on Meta analysis only prospective studies.)	prospective studies[18-20]							studies are not clear, so the causal relationship cannot be determined. Meanwhile, it requires a large amount of plasma/serum to detect
	IL-8	Unclear(Positive correlation based on Meta analysis irrespective of study types; Irrelevant based on prospective studies only.)	Meta analysis of 8 observational studies[12]+1 prospective study[18]	3a	ELISA	Serum or plasma	120.0μl	Yes[21]	No	The same reasons as IL-6.
	TNF-α	Positive	Meta analysis of 17 observational studies[21]	3a	ELISA	Serum or plasma	1.0μl	Yes[17, 21]	No	Not appropriate as a predictor of cancer, because it is a protein could secreted by tumor cells.

*Based on "Oxford Centre for Evidence-based Medicine – Levels of Evidence (March 2009)".The levels includes 1a, 1b, 1c, 2a, 2b, 2c, 3a, 3b, 4 and 5, where 1a is the systematic review of RCTs with the highest level of evidence. &Previous studies have detected these proteins using blood samples stored in refrigerator for years, which reflects the stability in vitro indirectly. E2, Estradiol; LEP, Leptin; sOB-R, Soluble leptin receptor; ADP, Adiponectin; RETN, Resistin; VF, Visfatin; IGF-1, Insulin-like growth factor 1; IGFBP-3, Insulin-like growth factor binding protein 3; CRP, C-reactive protein; IL-6, Interleukin- 6; IL-8, Interleukin- 8; TNF-α, Tumor necrosis factor-α.

Supplementary Table 2 The distribution and comparison of basic characteristics and risk factors among controls and cases of different menopausal status

	**Premenopausal**			**Postmenopausal**		
	**Cases(n=167)**	**Controls(n=149)**	**z/ χ^2 (*p* values)**	**Cases(n=112)**	**Controls(n=111)**	**z/ χ^2 (*p* values)**
Age (year)	44.00(41.00, 47.00)	44.00(41.00, 47.00)	-0.18(0.858)	57.50(53.00, 62.00)	56.00(52.00, 62.00)	-1.18(0.239)
Age at menopause (year)[&]	-	-	-	50.00(47.00, 51.00)	50.00(48.00, 52.00)	-1.02(0.310)
BMI(kg/m^2)	22.94(21.19, 24.80)	23.24(21.10, 24.99)	-0.67(0.504)	22.95(21.26, 25.07)	23.53(22.10, 25.11)	-1.38(0.167)
BMI at 5 years ago (kg/m^2)	22.58(20.81, 23.94)	22.35(20.42, 24.14)	-0.38(0.707)	23.12(21.22, 25.25)	23.23(21.83, 25.15)	-0.74(0.460)
BMI at 10 years ago (kg/m^2)	21.91(20.00, 24.00)	21.60(20.08, 23.43)	-0.86(0.389)	22.22(21.13, 24.53)	22.89(21.33, 25.00)	-0.58(0.559)
BMI at 20 years old (kg/m^2)	20.81(19.15, 22.04)	20.32(18.93, 22.13)	-1.17(0.242)	20.83(19.50, 22.64)	21.37(19.56, 22.83)	-0.71(0.476)
Energy intake (kcal)	1767.11(1494.57, 2213.43)	1501.09(1191.63, 1875.10)	**-4.92(<0.001)***	1835.40(1477.65, 2322.56)	1612.08(1287.83, 1978.40)	**-3.51(<0.001)***
WHR	0.81(0.74, 0.86)	0.83(0.79, 0.87)	**-3.43(0.001)***	0.83(0.76, 0.88)	0.86(0.82, 0.90)	**-3.52(<0.001)***
Age at menarche (year)	13.00(12.00, 14.00)	14.00(13.00, 15.00)	**-1.99(0.047)***	15.00(13.00, 16.00)	14.00(13.00, 16.00)	-1.46(0.144)
Duration of menstruation (year)[1]	31.00(27.00, 34.00)	30.00(27.00, 33.50)	-0.75(0.453)	34.00(32.00, 37.00)	35.00(33.00, 3700)	-1.52(0.128)
Age interval between menarche and first live birth (year)[$]	11.00(8.00, 13.00)	10.00(8.00, 13.00)	-1.46(0.145)	10.00(7.00, 12.00)	10.00(8.00, 12.00)	-0.89(0.375)
Age at first live birth (year)[$]	24.00(22.00, 26.00)	24.00(22.00, 26.00)	-0.66(0.509)	24.00(23.00, 26.00)	24.00(23.00, 26.25)	-0.42(0.677)
Residence			**14.72(0.002)***			**10.36(0.016)***
Urban	65(38.9%)	61(40.9%)		43(38.4%)	52(46.8%)	
County	31(18.6%)	8(5.4%)		18(16.1%)	4(3.6%)	
Town	20(12.0%)	30(20.1%)		14(12.5%)	12(10.8%)	
Rural	51(30.5%)	50(33.6%)		37(33.0%)	43(38.7%)	

Supplementary Table 2 The distribution and comparison of basic characteristics and risk factors among controls and cases of different menopausal status

	**Premenopausal**			**Postmenopausal**		
	**Cases(n=167)**	**Controls(n=149)**	**z/ χ^2 (*p* values)**	**Cases(n=112)**	**Controls(n=111)**	**z/ χ^2 (*p* values)**
Education			**26.82(<0.001)***			**15.00(0.010)***
Never enrolled or did not complete primary school	4(2.4%)	1(0.7%)		17(15.2%)	17(15.3%)	
Primary school	30(18.0%)	42(28.2%)		25(22.3%)	24(21.6%)	
Junior high school	67(40.1%)	46(30.9%)		33(29.5%)	21(18.9%)	
High school or technical secondary school	35(21.0%)	13(8.7%)		24(21.4%)	24(21.6%)	
College	20(12.0%)	16(10.7%)		11(9.8%)	8(7.2%)	
Bachelor degree or above	11(6.6%)	31(20.8%)		2(1.8%)	17(15.3%)	
Income(CNY)			**13.29(0.010)***			**24.92(<0.001)***
＜10000	16(9.6%)	8(5.4%)		21(18.8%)	4(3.6%)	
10000-29999	50(29.9%)	58(38.9%)		33(29.5%)	42(37.8%)	
30000-49999	45(26.9%)	31(20.8%)		33(29.5%)	21(18.9%)	
50000-99999	42(25.1%)	25(16.8%)		22(19.6%)	28(25.2%)	
＞100000	14(8.4%)	27(18.1%)		3(2.7%)	16(14.4%)	
Occupation			**12.28(0.015)***			**15.91(0.003)***
Unemployed	40(24.0%)	23(15.4%)		15(13.4%)	25(22.5%)	
Government, enterprises and public institutions	36(21.6%)	50(33.6%)		29(25.9%)	42(37.8%)	
Manufacturing, worker	29(17.4%)	15(10.1%)		19(17.0%)	4(3.6%)	
Business, services	44(26.3%)	36(24.2%)		15(13.4%)	10(9.0%)	
Agricultural	18(10.8%)	25(16.8%)		34(30.4%)	30(27.0%)	
Smoking			0.07(0.786)			2.26(0.132)
No	163(97.6%)	147(98.7%)		108(96.4%)	111(100.0%)	

Supplementary Table 2 The distribution and comparison of basic characteristics and risk factors among controls and cases of different menopausal status

	**Premenopausal**			**Postmenopausal**		
	**Cases(n=167)**	**Controls(n=149)**	**z/ χ^2 (p values)**	**Cases(n=112)**	**Controls(n=111)**	**z/ χ^2 (p values)**
Yes	4(2.4%)	2(1.3%)		4(3.6%)	0(0.0%)	
Passive smoking			0.11(0.735)			**3.31(0.069)***
No	82(49.1%)	76(51.0%)		54(48.2%)	67(60.4%)	
Yes	85(50.9%)	73(49.0%)		58(51.8%)	44(39.6%)	
Alcohol			0.72(0.395)			0.12(0.728)
No	164(98.2%)	143(96.0%)		107(95.5%)	108(97.3%)	
Yes	3(1.8%)	6(4.0%)		5(4.5%)	3(2.7%)	
Exercise			**3.56(0.059)***			0.19(0.668)
No	132(79.0%)	104(69.8%)		99(88.4%)	96(86.5%)	
Yes	35(21.0%)	45(30.2%)		13(11.6%)	15(13.5%)	
Live birth			0.02(0.592)			0.80(0.371)
No	3(1.8%)	4(2.7%)		4(3.6%)	1(0.9%)	
Yes	164(98.2%)	145(97.3%)		108(96.4%)	110(99.1%)	
Lactation			0.23(0.633)			0.33(0.568)
No	16(9.6%)	12(8.1%)		16(14.3%)	13(11.7%)	
Yes	151(90.4%)	137(91.9%)		96(85.7%)	98(88.3%)	
Adverse pregnancy history[2]			**2.78(0.095)***			**3.05(0.081)***
No	151(90.4%)	142(95.3%)		100(89.3%)	106(95.5%)	
Yes	16(9.6%)	7(4.7%)		12(10.7%)	5(4.5%)	
Abortion[3]			**10.67(0.005)***			**8.11(0.017)***
No	31(18.6%)	30(20.1%)		29(25.9%)	37(33.3%)	
1 kind of	117(70.1%)	116(77.9%)		69(61.6%)	71(64.0%)	
≥2 kinds of	19(11.4%)	3(2.0%)		14(12.5%)	3(2.7%)	
Contraception[4]			4.78(0.189)			4.12(0.128)
No	54(32.3%)	44(29.5%)		36(32.1%)	39(35.1%)	
1 kind of	99(59.3%)	100(67.1%)		72(64.3%)	72(64.9%)	
2 kinds of	13(7.8%)	4(2.7%)		4(3.6%)	0(0.0%)	

Supplementary Table 2 The distribution and comparison of basic characteristics and risk factors among controls and cases of different menopausal status

	**Premenopausal**			**Postmenopausal**		
	**Cases(n=167)**	**Controls(n=149)**	**z/ χ^2 (*p* values)**	**Cases(n=112)**	**Controls(n=111)**	**z/ χ^2 (*p* values)**
⩾3 kinds of	1(0.6%)	1(0.7%)			0(0.0%)	
Perimenopausal symptoms			0.44(0.508)			0.11(0.745)
No	155(92.8%)	141(94.6%)		92(82.1%)	93(83.8%)	
Yes	12(7.2%)	8(5.4%)		20(17.9%)	18(16.2%)	
Hormone replacement therapy			0.40(0.529)			<0.001(1.00)
No	165(98.8%)	149(100.0%)		109(97.3%)	108(97.3%)	
Yes	2(1.2%)	0(0.0%)		3(2.7%)	3(2.7%)	
Cardiovascular disease[5]			<0.01(0.954)			<0.001(0.994)
No	167(100.0%)	148(99.3%)		111(99.1%)	109(98.2%)	
Yes	0(0.0%)	1(0.7%)		1(0.9%)	2(1.8%)	
Diabetes			<0.01(1.00)			0.07(0.788)
No	166(99.4%)	148(99.3%)		105(93.8%)	105(94.6%)	
Yes	1(0.6%)	1(0.7%)		7(6.3%)	6(5.4%)	
Breast/cervix/ovary disease			0.12(0.735)			**3.10(0.078)***
No	100(59.9%)	92(61.7%)		65(58.0%)	77(69.4%)	
Yes	67(40.1%)	57(38.3%)		47(42.0%)	34(30.6%)	
Family history of cardiovascular disease			1.22(0.270)			0.03(0.872)
No	130(77.8%)	108(72.5%)		95(84.8%)	95(85.6%)	
Yes	37(22.2%)	41(27.5%)		17(15.2%)	16(14.4%)	
Family history of diabetes			0.25(0.616)			1.26(0.262)
No	156(93.4%)	137(91.9%)		107(95.5%)	102(91.9%)	

Supplementary Table 2 The distribution and comparison of basic characteristics and risk factors among controls and cases of different menopausal status

	**Premenopausal**			**Postmenopausal**		
	**Cases(n=167)**	**Controls(n=149)**	**z/ χ^2 (*p* values)**	**Cases(n=112)**	**Controls(n=111)**	**z/ χ^2 (*p* values)**
Yes	11(6.6%)	12(8.1%)		5(4.5%)	9(8.1%)	
Family history of breast/cervical/ovarian cancer			1.27(0.261)			1.37(0.242)
No	154(92.2%)	142(95.3%)		104(92.9%)	107(96.4%)	
Yes	13(7.8%)	7(4.7%)		8(7.1%)	4(3.6%)	

Quantitative variables were described as the median (25%, 75%) and rank sum test was used. Classification variables were described as frequency (composition ratio) and chi-square test was used. [1.] Duration of menstruation was equal to age minus age of menarche for premenopausal women, while equal to age at menopause minus age of menarche for postmenopausal women. [2]Adverse pregnancy history included ectopic pregnancy, fetal development arrest, premature delivery and threatened abortion. [3]Abortion included spontaneous abortion, induced abortion, drug abortion and induced labor. [4]Contraceptive included intrauterine contraceptive device, long-acting contraceptive pill, short-acting contraceptive pill and emergency contraceptive pill. [5]Cardiovascular diseases included hypertension and coronary heart disease. [$]Participants who met any one of the following three criteria were diagnosed with menopause: ① aged 50 or older; ② underwent bilateral oophorectomy or surgical sterilization; and ③ younger than 50 years old, but menstrual periods has been absent for at least 12 months. *p<0.10. [$]There was missing (4 controls and 3 cases were missing among premenopausal subjects; 2 controls and 4 cases were missing among postmenopausal subjects)

Supplementary Table 3 The weight coefficients of standardized proteins of different menopausal status based on random forest model

Status	Proteins	Weight coefficients	Proportion
Premenopausal	RETN	19.10609	15%
	ADP	18.00972	14%
	IGF-1	16.79015	13%
	IGFBP-3	16.44989	13%
	CRP	14.76103	12%
	sOB-R	14.3512	11%
	LEP	13.86147	11%
	VF	12.77187	10%
Postmenopausal	ADP	18.441782	28%
	RETN	7.788563	12%
	IGF-1	6.804443	10%
	CRP	6.191498	9%
	E2	6.169918	9%
	VF	6.074258	9%
	IGFBP-3	5.963165	9%
	sOB-R	5.688581	8%
	LEP	3.928103	6%

ADP, Adiponectin; RETN, Resistin; IGF-1, Insulin-like growth factor 1; VF, Visfatin; sOB-R, Soluble leptin receptor; IGFBP-3, Insulin-like growth factor binding protein 3; CRP, C-reactive protein; LEP, Leptin; E2, Estradiol.

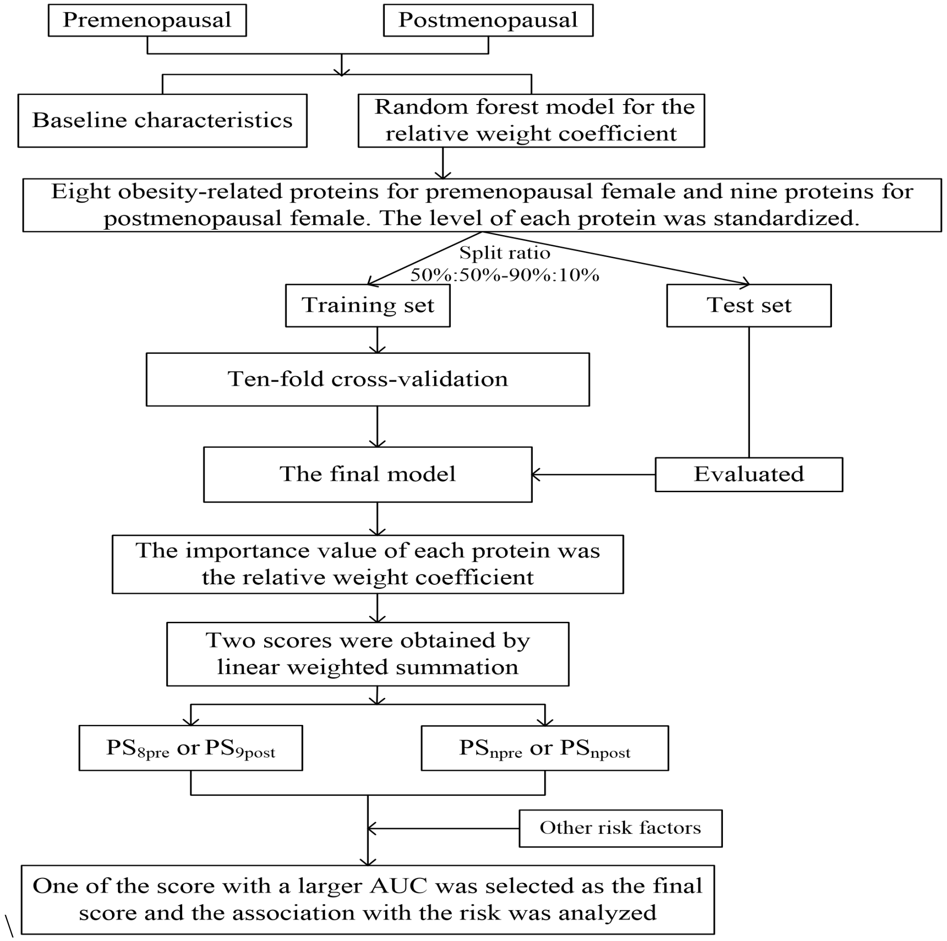

Supplementary Fig.1 An overview of the analysis process

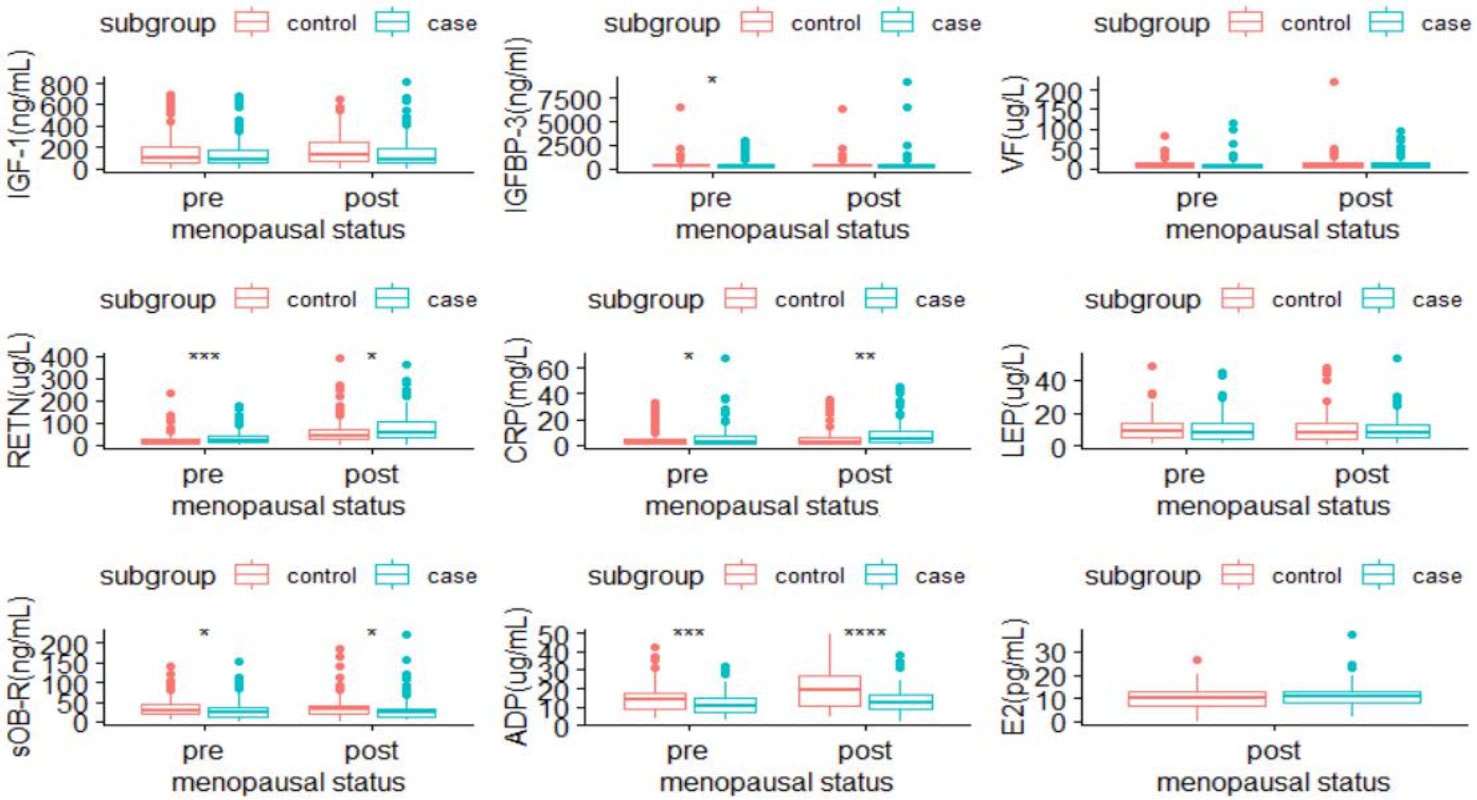

Supplementary Fig. 2 The boxplots of proteins among cases and controls of different menopausal status

*$p \leqslant 0.05$; **$p \leqslant 0.01$; ***$p \leqslant 0.001$; ****$p \leqslant 0.0001$; pre, premenopausal female; post, postmenopausal female

The curve was conducted using R 3.5.1 software (R Core Team (2018), Vienna, Austria. URL: https://www.R-project.org/)

Reference

[1] Walker K., Bratton D.J., Frost C. Premenopausal endogenous oestrogen levels and breast cancer risk: a meta-analysis[J]. British journal of cancer,2011,**105**(9):1451-1457.

[2] Bolelli G., Muti P., Micheli A., Sciajno R., Franceschetti F., Krogh V., et al. Validity for Epidemiological Studies Steroid and Protein Hormones of Long-Term Cryoconservation in Serum and Plasma[J]. Cancer Epidemiol Biomarkers Prev,1995,**4**(5):509-513.

[3] Kley H.K., Schlaghecke R., Krüskemper H.L. Stability of steroids in plasma over a 10-year period[J]. J Clin Chem Clin Biochem,1985,**23**(12):875-878.

[4] Key T., Appleby P., Barnes I., Reeves G. Endogenous sex hormones and breast cancer in postmenopausal women: reanalysis of nine prospective studies[J]. J Natl Cancer Inst,2002,**94**(8):606-616.

[5] Pan H., Deng L.L., Cui J.Q., Shi L., Yang Y.C., Luo J.H., et al. Association between serum leptin levels and breast cancer risk: An updated systematic review and meta-analysis[J]. Medicine (Baltimore),2018,**97**(27):e11345.

[6] Evans M.J., Livesey J.H., Ellis M.J., Yandle G.T. Effect of anticoagulants and storage temperatures on stability of plasma and serum hormones[J]. Clin Biochem,2001,**34**(2):107-112.

[7] Rodrigo C., Tennekoon K.H., Karunanayake E.H., Silva K.D., Amarasinghe T., Wijayasiri A. Circulating leptin, soluble leptin receptor, free leptin index, visfatin and selected leptin and leptin receptor gene polymorphisms in sporadic breast cancer[J]. Endocr J,2017,**64**(4):393-401.

[8] Mohammadzadeh G., Ghaffari M.A., Bafandeh A., Hosseini S.M. Association of serum soluble leptin receptor and leptin levels with breast cancer[J]. J Res Med Sci,2014,19(5):433-438.

[9] Zeman M. Serum Adiponectin Relates to Shortened Overall Survival in Men with Squamous Cell Esophageal Cancer Treated with Preoperative Concurrent Chemoradiotherapy: A Pilot Study[J]. Medical Science Monitor,2014,**20**:2351-2357.

[10] Gu L., Cao C., Fu J., Li Q., Li D.H., Chen M.Y. Serum adiponectin in breast cancer: A meta-analysis[J]. Medicine (Baltimore),2018,**97**(29):e11433.

[11] Wang Z.M., Gao S, Sun C.X., Li J.Y., Gao W.H., Yu L.P. Clinical significance of serum adiponectin and visfatin levels in endometrial cancer[J]. International Journal of Gynecology & Obstetrics,2019,**14**5(1):34-39.

[12] Gui Y., Pan Q.W., Chen X.C., Xu S.M., Luo X.D., Chen L. The association between obesity related adipokines and risk of breast cancer: a meta-analysis[J]. Oncotarget,2017,**8**(43):75389-75399.

[13] Mahieu M.A., Ahn G.E., Chmiel J.S., Dunlop D.D., Helenowski I.B., Semanik P., et al. Serum adipokine levels and associations with patient-reported fatigue in systemic lupus erythematosus[J]. Rheumatology International,2018,**38**(6):1053-1061.

[14] Key T.J., Appleby P.N., Reeves G.K., Roddam A.W. Insulin-like growth factor 1 (IGF1), IGF binding protein 3 (IGFBP3), and breast cancer risk: pooled individual data analysis of 17 prospective studies[J]. Lancet Oncol,2010,**11**(6):530-542.

[15] Hernandez A.V., Guarnizo M., Miranda Y., Pasupuleti V., Deshpande A., Paico S., et al. Association between insulin resistance and breast carcinoma: a systematic review and meta-analysis[J]. PLoS One,2014,**9**(6):e99317.

[16] Chan D.S., Bandera E.V., Greenwood D.C., Norat T. Circulating C-Reactive Protein and Breast Cancer Risk-Systematic Literature Review and Meta-analysis of Prospective Cohort Studies[J]. Cancer Epidemiol Biomarkers Prev,2015,**24**(10):1439-1449.

[17] Graham C., Chooniedass R., Stefura W.P., Lotoski L., Lopez P., Befus A.D, et al. Stability of pro- and anti-inflammatory immune

biomarkers for human cohort studies[J]. J Transl Med,2017,**15**(1):53.

[18] Dias J.A., Fredrikson G.N., Ericson U, Gullberg B., Hedblad B., Engström G., et al. Low-Grade Inflammation, Oxidative Stress and Risk of Invasive Post-Menopausal Breast Cancer - A Nested Case-Control Study from the Malmö Diet and Cancer Cohort[J]. PLOS ONE,2016,**11**(7):e158959.

[19] Agnoli C, Grioni S, Pala V, Allione A., Matullo G., Gaetano C.D., et al. Biomarkers of inflammation and breast cancer risk: a case-control study nested in the EPIC-Varese cohort[J]. Scientific Reports,2017,**7**(1).

[20] Gunter M.J., Wang T, Cushman M, Xue X.Y., Smoller S.W., Strickler H.D., et al. Circulating Adipokines and Inflammatory Markers and Postmenopausal Breast Cancer Risk[J]. JNCI: Journal of the National Cancer Institute,2015,**107**(9).

[21] Friebe A., Volk H. Stability of Tumor Necrosis Factor α, Interleukin 6, and Interleukin 8 in Blood Samples of Patients With Systemic Immune Activation[J]. Arch Pathol Lab Med,2008,**11**(132):1802-1806.
